# Role of imbalanced gut microbiota in promoting CRC metastasis: from theory to clinical application

**DOI:** 10.1186/s12964-024-01615-9

**Published:** 2024-04-18

**Authors:** Shiying Fan, Lujia Zhou, Wenjie Zhang, Daorong Wang, Dong Tang

**Affiliations:** 1 Clinical Medical College, Yangzhou University, 225000 Yangzhou, P. R. China; 2 School of Medicine, Chongqing University, 400030 Chongqing, P. R. China; 3 Department of General Surgery, Institute of General Surgery, Clinical Medical College, Northern Jiangsu People’s Hospital, Yangzhou University, 225000 Yangzhou, P. R. China

**Keywords:** Colorectal cancer, Gut microbiota, Metastasis, “seed and soil” hypothesis

## Abstract

Metastasis poses a major challenge in colorectal cancer (CRC) treatment and remains a primary cause of mortality among patients with CRC. Recent investigations have elucidated the involvement of disrupted gut microbiota homeostasis in various facets of CRC metastasis, exerting a pivotal influence in shaping the metastatic microenvironment, triggering epithelial-mesenchymal transition (EMT), and so on. Moreover, therapeutic interventions targeting the gut microbiota demonstrate promise in enhancing the efficacy of conventional treatments for metastatic CRC (mCRC), presenting novel avenues for mCRC clinical management. Grounded in the “seed and soil” hypothesis, this review consolidates insights into the mechanisms by which imbalanced gut microbiota promotes mCRC and highlights recent strides in leveraging gut microbiota modulation for the clinical prevention and treatment of mCRC. Emphasis is placed on the considerable potential of manipulating gut microbiota within clinical settings for managing mCRC.

## Introduction

Colorectal cancer (CRC) ranks as the third most prevalent and second most deadly cancer worldwide [1]. It is estimated that 25% of patients with CRC present with distant metastasis at their initial diagnosis, while nearly 50% develop metastasis during the disease course [2]. Metastasis, a hallmark of tumour cells, signifies the detachment of tumour cells from the primary lesion, their migration to distant organs or tissues, and the establishment of secondary tumours through modes such as direct spread, lymphatic metastasis, haematogenous dissemination, and implantation, representing the most severe manifestation of cancer [3]. Among metastatic sites, the liver is the most frequent, accounting for approximately 60% of all metastatic colorectal cancer (mCRC) cases [4]. Despite advancements in treatment, mCRC continues to pose substantial lethality, with a 5-year survival rate of approximately 14% [5, 6]. Therefore, the identification of effective treatment options to enhance the survival and quality of life of patients with mCRC is imperative. Presently, the main therapeutic strategy for unresectable mCRC is systemic therapy, including chemotherapy, immunotherapy, targeted therapy, and their combinations. However, drug resistance remains a primary impediment to the efficacy of these therapeutic strategies [7].

The gut microbiota in CRC patients has been found to be significantly imbalanced and different from the normal population [8, 9]. Under normal circumstances, the gut microbiota maintains homeostasis by metabolizing indigestible dietary components, synthesizing nutrients such as vitamins, detoxifying metabolites, modulating immune responses, facilitating epithelial cell renewal, preserving mucosal integrity, and producing antimicrobial compounds, thereby contributing to colonic function and human health [10]. However, alterations in the abundance of the healthy gut microbiota can foster chronic inflammation and the generation of carcinogenic metabolites, predisposing to tumorigenesis [10]. Given the altered gut microbiota in CRC, researchers have highlighted its pivotal role in CRC development and progression. Disruption of gut microbial equilibrium promotes CRC progression, with the imbalanced gut microbiota emerging as a significant contributor to CRC metastasis [11]. Current evidence indicates that the gut microbiota modulates various processes involved in CRC metastasis, including epithelial-mesenchymal transition (EMT), thereby facilitating the migration and invasion of CRC cells [12]. Furthermore, the gut microbiota influences the efficacy of clinical interventions for mCRC [13]. Despite an incomplete understanding of the precise mechanisms underlying the action of the gut microbiota in mCRC development, its crucial involvement in tumour metastasis and clinical outcomes continues to garner substantial attention. This review aims to elucidate the mechanisms through which the gut microbiota contributes to CRC metastasis and proposes potential clinical implications of targeting the gut microbiota in the prevention, adjunctive therapy, and prognostication of mCRC, thereby offering insights for both theoretical understanding and clinical practice in managing patients with mCRC.

## CRC metastasis

CRC metastasis can affect multiple neighbouring or distant organs and tissues, including the liver, lungs, bones, and brain, thereby causing damage and leading to a decline in the quality of life and even death among patients with mCRC [14]. Hence, it is imperative to elucidate the pathways and intricate mechanisms underlying CRC metastasis to impede its progression and enhance the prognosis of patients with CRC. Over the past few decades, scientists have endeavoured to unravel the fundamental cellular and molecular mechanisms responsible for CRC metastasis, achieving significant breakthroughs and progress. They have not only elucidated the various pathways of CRC metastasis but have also proposed the metastatic cascade and the “seed and soil” hypothesis of mCRC, thereby deepening our comprehension of CRC metastasis [15, 16].

### CRC metastasis routes

CRC commonly metastasizes through various routes, including direct spread, lymphatic metastasis, hematogenous dissemination, and implantation dissemination [15, 17]. Lymphatic metastasis and hematogenous dissemination stand out as the primary mechanisms of CRC metastasis [4]. The lymphatic system, an integral part of the circulatory system, comprises capillary lymphatic vessels that pervade nearly every tissue in the body. These vessels typically feature a single layer of endothelial cells, characterized by large intercellular gaps and loose cellular connections, facilitating the traversal of tumour cells from the vessel wall into the lumen and subsequent metastasis to distant sites [18]. Factors governing lymph angiogenesis and lymphatic vessel remodelling correlate with cancer progression in patients [18]. Hematogenous dissemination entails the infiltration of tumour cells into blood vessels and their subsequent dissemination to distant sites through the bloodstream, often manifesting in the liver [19]. This predilection for hepatic metastasis can be attributed to the unique vascular architecture of the liver, with its dual blood supply from the hepatic portal vein and hepatic artery, affording ample opportunities for circulating cancer cells to colonize this organ. This phenomenon underscores the predominant pattern of metastases from primary tumours to specific secondary organs [20]. Furthermore, features such as sluggish blood flow velocities, heightened permeability of liver sinusoidal endothelial cells (LSECs), and the expression of adhesion molecule-driven docking signals facilitate the infiltration of disseminated cancer cells [20, 21]. Additionally, the immune tolerance capacity of the liver shapes the immunosuppressive microenvironment, thereby mitigating excessive immune responses to antigens entering the liver and averting the depletion of cancer cells [22]. The liver is also rich in growth factors such as epidermal growth factor (EGF) and vascular endothelial growth factor (VEGF), which have a positive correlation with the liver colonization potential of CRC, and can stimulate metastasis [23, 24]. These characteristics inherently make the liver susceptible to haematogenous spread. Additionally, direct spread offers another route for CRC cells to invade adjacent tissues or organs [17, 25]. The right side of the colon, especially the hepatic flexure of the colon, is adjacent to the liver, which can lead to direct spread of CRC cells to the liver, another reason why the liver is a common metastatic target organ of CRC [26]. It is worth noting that, for these various reasons, the common metastatic site of most types of CRC is the liver, but there are also some rare types of CRC, such as primary colorectal lymphoma and gastrointestinal mesenchymal tumours, in which the common metastatic sites are the spleen, peritoneum, etc [4]. This variation in metastatic sites may be attributed to tumour-specific characteristics, although comprehensive studies are lacking. Furthermore, the term “implantation dissemination” describes the process wherein single or clustered tumour cells detach from the primary tumour and enter the peritoneal cavity. These free tumour cells adhere to the distant peritoneum and invade the subperitoneal space, where the underlying connective tissues provide the necessary scaffolding and angiogenesis to support tumour proliferation and further metastatic growth. This process ultimately leads to peritoneal carcinomatosis [15]. When cancer cells disseminate widely in the peritoneum, a large volume of malignant ascites may develop, resulting from increased permeability due to the obstruction of lymphatic or blood vessels within the peritoneal cavity and secondary hypoalbuminemia [27]. Malignant ascites harbour elevated levels of immunosuppressive cytokines, further contributing to the refractory nature of CRC peritoneal metastasis [28]. In summary, the metastatic pathways of CRC are diverse, and influenced by anatomical, histological, physiological, and immunological factors, leading to varied metastatic outcomes. Therefore, elucidating the specific metastatic mechanisms of CRC is crucial.

### CRC metastasis mechanisms

**Fig. 1:**
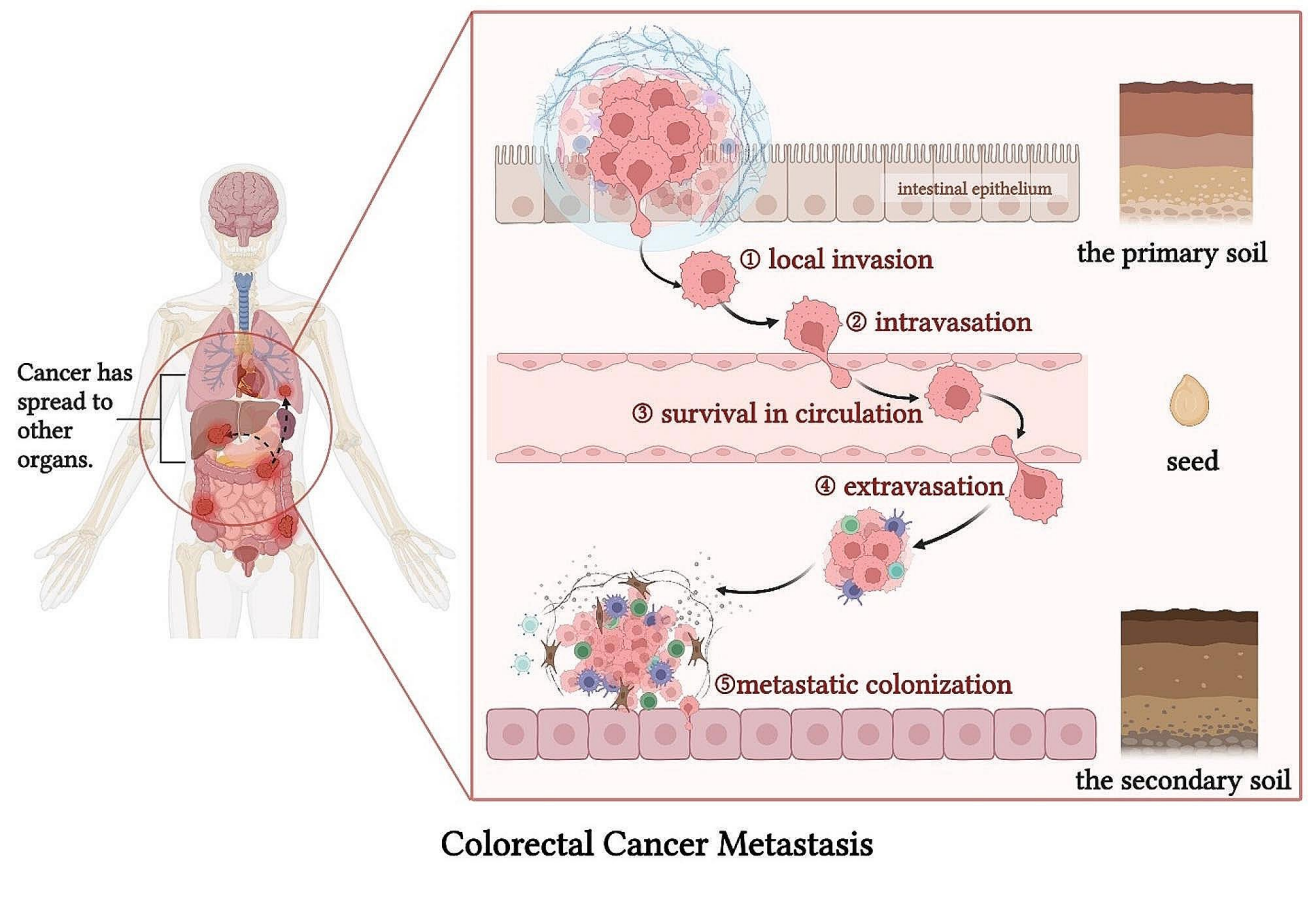
The process of colorectal cancer metastasis. Colorectal cancer cells undergo local invasion, intravasation, survival in circulation, extravasation, and metastatic colonization to achieve distant metastasis. The Figure was created with BioRender.com

The process of CRC metastasis can be succinctly divided into several steps: cellular migration into the surrounding tissue [local invasion], diffusion into the vasculature [intravasation], continued metastasis of circulating tumour cells (CTCs) within the circulatory system and their subsequent survival [survival in circulation], escape of CTCs from the vasculature [extravasation], invasion into surrounding tissues, and settlement and proliferation at a new site [metastatic colonization]. These steps collectively constitute the invasion-metastasis cascade [3, 16]. The “seed and soil” hypothesis is the classic theory of metastasis in malignant tumours such as CRC: in the metastatic cascade, tumour cells with metastatic potential [seed] invade, detach from the primary tumour microenvironment [the primary soil] after proliferating in it, and then enter the circulatory system and disperse throughout the body. Even before the seeds are detached, the primary soil is constantly transforming distant metastatic target organs [the secondary soil] through exosomes, cytokines, etc. The secondary soil that supports the growth of the seeds in the circulatory system is where the seeds prefer to settle [29]. Clearly, CRC metastasis involves a complex chain reaction comprising various biological processes (Fig. 1). While the fundamental framework of the CRC metastasis mechanism has been established, the numerous signaling pathways involved in metastasis remain incompletely elucidated, presenting a challenge for future research endeavours.

## Correlation of gut microbiota alterations with CRC metastasis

There is now some clinical evidence and animal studies suggesting that CRC metastasis may be influenced by alterations in the gut microbiota. Two cohort studies by metagenomic analysis of fecal or mucosal biopsies revealed marked changes in the composition of the gut microbiota in subjects ranging from adenomatous polyps to advanced CRC: the abundance of *Fusobacterium*, *Bacteroides fragilis* (*B. fragilis*), *Gemella*, and *Parvimonas* consistently increased with the progression of malignancy, while certain beneficial microorganisms, such as *Faecalibacterium prausnitzii* (*F. prausnitzii*), were depleted in the gut of subjects with advanced CRC [30, 31]. Furthermore, *Fusobacterium nucleatum* (*F. nucleatum*) was not only present in primary colorectal tumours, but its colonization was maintained in distant metastasis, such as the liver, demonstrating the stability of the microbiota between paired primary and metastatic tumours and implying that distant metastasis of CRC may be accompanied by the migration of *F. nucleatum* [32]. CRC liver metastasis (CRLM) was significantly enhanced when mice were orally administered *F. nucleatum*, which was associated with sustained exposure to *F. nucleatum*, leading to a reduction in the diversity of the gut microbiota in mice and inducing an imbalance in the gut microbiota [33]. In vivo experiments have also found that *Escherichia coli* (*E. coli*) can promote the metastasis of CRC cells to the liver [34]. Although the antibiotic vancomycin can kill Gram-positive bacteria, it is ineffective against the Gram-negative bacteria *F. nucleatum* and *E. coli*, and can even increase their proportion in the intestines of healthy people, destroying the original intestinal environment [35, 36]. The vancomycin treatment of mice inoculated with colon tumour cells via the spleen was found to enhance CRLM in mice, which is largely due to the disruption of the original balanced gut microbiota [37]. The disordered gut microbiota is manifested in an increased abundance of *Parabacteroides distasonis* and *Proteus mirabilis*, and elevated populations of them might favor the CRLM [37]. These animal experiments illustrate that elevated levels of tumour-invasive pathogens are closely linked to mCRC, and that the gut ecological dysregulation enhances distant metastasis.

Taken together, the composition of the gut microbiota of CRC patients is quite different from that of the healthy population, and further in vivo evidence suggests that the gut ecological dysregulation is a pathogenic factor that promotes the progression and metastasis of CRC. However, the results of the in vivo experiments described in this section only illustrate the relativity between the gut microbiota and CRC metastasis: the imbalanced gut microbiota is closely correlated with the progression of mCRC. Therefore, further attention to the specific mechanisms by which the imbalanced gut microbiota promotes mCRC is the focus of our current research, which may provide novel approaches to the prevention and treatment of mCRC. The relevant mechanisms will be described in detail in the next section, including but not limited to: promoting EMT, changing the primary tumour microenvironment (TME), inducing the formation of pre-metastatic niche (PMN), and so on.

## Mechanisms underlying the promotion of CRC metastasis by imbalanced gut microbiota

An increasing number of in vivo and in vitro experiments have delved into the mechanisms through which imbalanced gut microbiota contributes to CRC metastasis. Significant progress has been achieved, offering fresh insights into the association between CRC metastasis and the gut microbiota [4]. According to the “seed and soil” hypothesis, CRC metastasis involves seeds, the primary soil and the secondary soil, and the disordered gut ecology can also modify these three aspects separately, thereby facilitating the onset of mCRC [33, 38, 39]. The mechanisms involved are further described below.

### Promoting EMT and cancer stem cells (CSCs) formation: targeting seeds

EMT is the process through which epithelial cells transform into mesenchymal cells, enhancing their mobility and invasive potential, thereby facilitating their proximity to blood vessels and subsequent dissemination into circulation [40]. Specifically, tumour cells in the front line of invasive tumours typically lose epithelial markers and intercellular adhesions as well as express more mesenchymal markers [29]. Gradually, CRC cells change into a mesenchymal phenotype that is more invasive, motile, and metastatic, and the increased harmful pathogenic bacteria in the imbalanced gut microbiota can promote the occurrence of this process by upregulating EMT-related regulators [12, 41]. *F. nucleatum* is a pathogenic bacterium that has been extensively studied in relation to the EMT process in CRC cells. *F. nucleatum* can activate specialized surface proteins expressed on the membrane of immune cells (including neutrophils), Toll-like receptor 4 (TLR4), to promote the production of reactive oxygen species (ROS), or directly activate NOD-like receptor 1/2 (NLR1/2) signaling, and therefore inducing the formation and release of extensive neutrophil extracellular traps (NETs) from neutrophils [42]. Subsequently, CRC cells that are captured by NETs express lower levels of the epithelial marker, E-cadherin, and express higher levels of mesenchymal markers, N-cadherin and Vimentin, to promote EMT [42]. The epidermal growth factor receptor (EGFR), one of the tyrosine kinases associated with EMT, and the phosphorylation of its downstream effector kinases, including protein kinase B (AKT) and extracellular signal-regulated kinase (ERK), can also be induced and enhanced by *F. nucleatum*, thereby activating the EMT process in CRC cells [43]. Besides, *F. nucleatum* was found to produce a novel virulence molecule, DNA hunger/stationary phase protective proteins (Dps), which stimulates macrophages to secrete chemokines C-C motif chemokine ligand (CCL) 2/7, thus regulating the expression of relevant factors involved in the EMT process to promote tumour metastasis [44]. In addition, *F. nucleatum* also regulates the expression of non-coding RNAs, including long non-coding RNAs (lncRNAs) and microRNAs (miRNAs), to mediate the EMT process in CRC cells [45, 46]. LncRNAs are RNAs longer than 200 nucleotides, and miRNAs are 20- to 22-nucleotide-single-stranded RNAs with a highly stable structure [47, 48]. Both of them can regulate critical cellular processes, such as proliferation, apoptosis, differentiation, and metabolism, as well as regulate physiological and pathological processes such as cell cycle and DNA damage repair, whose disruption can result in the progression of malignant tumours [48, 49]. The mechanisms through which *F. nucleatum* affects EMT by modulating lncRNAs and miRNAs have progressively come to light. *F. nucleatum* upregulates the expression of the endogenous retroviral-associated adenocarcinoma lncRNA (*EVADR*), and then makes the elevated *EVADR* a modular scaffold of Y-box binding protein 1 (YBX1), an RNA-binding protein that is capable of stimulating the production of EMT-associated factors, to directly enhances the translation of EMT-associated factors, such as Snail, Slug and Zeb1, thereby inducing EMT [45, 50]. *F. nucleatum* infection reduces the level of the intracellular tumour suppressor gene miR-122-5p to cause overexpression of its downstream target α1,6-Fucosyltransferase (FUT8), which activates the transforming growth factor-β1 (TGF-β1)/Smads signaling pathway, inducing a decrease in E-cadherin levels while contributing to an increase in N-cadherin, Vimentin, Snail and Slug levels to promote EMT [46]. However, it is widely acknowledged that individual RNA is not expected to have a significant impact on CRC progression. Other potentially upregulated or downregulated RNAs induced by *F. nucleatum* may also have an impact on the EMT process in CRC cells. These issues are future studies that will need to try to address. Based on the important role of *F. nucleatum* in promoting EMT, it was found that treatment with metronidazole significantly reduced *F. nucleatum* load and inhibiting mCRC [51]. In addition to *F. nucleatum* inducing the EMT process in CRC cells, some other pathogenic bacteria, such as Enterotoxigenic *B. fragilis* (ETBF), *E. coli*, *Salmonella enteric* (*S. enterica*), etc., can also promote this process through various mechanisms [12]. However, the mechanisms underlying the promotion of EMT by these microorganisms in CRC cells remain poorly understood, and many links in the mechanistic pathways require validation through in vivo or in vitro experiments.

As mentioned previously, tumour cells that undergo EMT typically exhibit mesenchymal characteristics, stem cell-like properties, and enhanced migratory capacity, which enable them to infiltrate into the circulation and spread, and such cells are referred to as circulating tumour cells (CTCs) [29]. Upon entry into the vascular system, CTCs are exposed to the attack of hemodynamic forces, oxidative stress, immune cells, and other substances, so they adhere to platelets or form CTC clusters to protect themselves from damage to survive, leading to extravasate and colonize in distant organs [52, 53]. According to the “seed and soil” hypothesis, CTCs are the seeds that are separated from the primary soil and have the potential to grow in the secondary soil [54]. Although there is no definitive study evaluating the direct effect of the gut microbiota on CTCs, we reviewed in the previous section the role of the gut microbiota in inducing EMT, a biological process that is closely related to the migration and viability of CTCs, as it was found that common CTCs have less invasion, migration, and tumour immune escape compared to CTCs that have received EMT [55]. Hence, there likely exists a potential interaction between dysregulated gut microbiota and CTC behaviour, albeit this hypothesis remains unverified.

Moreover, CSCs are present in CRC tissues. CSCs are cancer cells with stem cell properties such as self-replication and multicellular differentiation, which are responsible for CRC recurrence and metastasis [56, 57]. During CRC metastasis, a few surviving CTC subpopulations have the CSCs phenotype [58]. This suggests that CSCs can be regarded as seeds with optimal chances of survival and colonization in the secondary soil. It has been found that certain oncogenic pathogens can target CSCs with metastatic potential to promote CRC metastasis. For instance, *F. nucleatum* binds to the bacterial receptor carcinoembryonic antigen-related cell adhesion molecule 1 (CEACAM-1) via the protein CbpF, leading to the dissociation of CEACAM-1 from its associated cytoplasmic tyrosine phosphatase SHP-2 to trigger a growth factor-like signaling cascade in CSCs to enhance CSCs stemness and then promote CRC reactivation and metastasis [57]. The CRC-associated microbiota metabolite isovalerate (IVA) initiates transcription of the rate-limiting enzyme tryptophan hydroxylase 2 (Tph2) to synthesize gut 5-hydroxytryptamine (5-HT), and then 5-HT triggers Wnt/β-catenin signaling by engagement with 5-HT receptor (HTR) 1B / 1D / 1 F on colorectal CSCs, promoting the self-renewal and metastasis of colorectal CSCs [59].

**Fig. 2:**
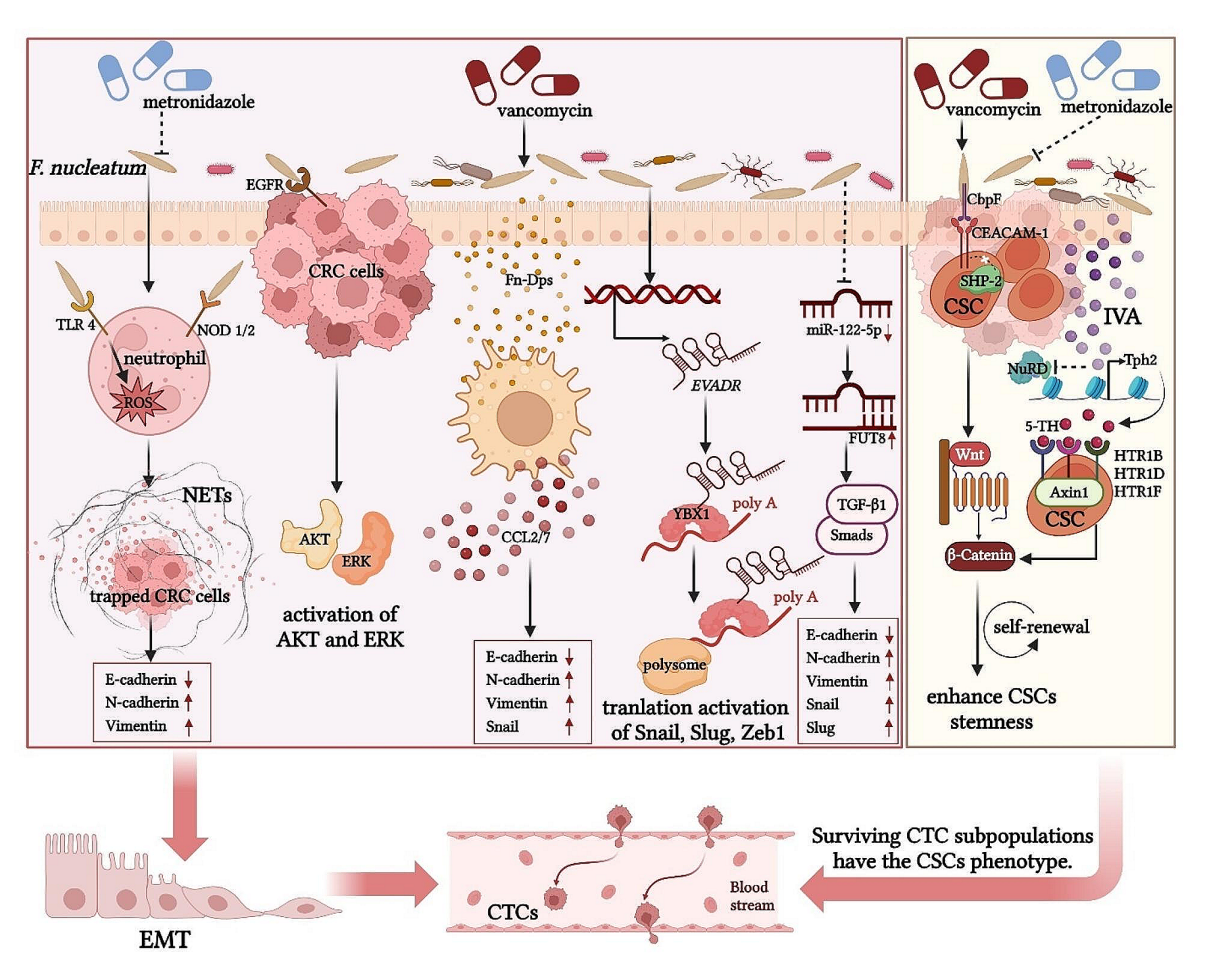
The mechanisms by which the gut microbiota promotes CRC metastasis by targeting seeds. (1) Promote the EMT process: *F. nucleatum* induces the formation and release of NETs from neutrophils by activating TLR4-ROS signaling and NLR1/2, and with consequent reduced expression of E-cadherin as well as increased expression of N-cadherin and vimentin in CRC cells captured by NETs. *F. nucleatum* also activates the downstream effector kinases AKT and ERK via EGFR. Fn-Dps, a novel virulence molecule produced by *F. nucleatum*, stimulates macrophages to secrete CCL2/7, thereby regulating the expression of related factors involved in EMT. *F. nucleatum* upregulates *EVADR* levels, which guide the RNA-binding protein YBX1 to recruit Snail, Slug, and Zeb1, to polysomes. *F. Nucleatum* infection decreases the level of the miR-122-5p, resulting in the overexpression of FUT8 to activate TGF-β1/Smads signaling pathway, inducing a decrease in E-cadherin levels and an increase in N-cadherin, Vimentin, Snail and Slug levels. (2) Promote the self-renewal of CSCs to enhance the stemness: *F. nucleatum* binds to CEACAM-1 on CSCs via CbpF to cause the dissociation of CEACAM-1 from SHP-2, triggering Wnt/β-catenin signaling. The gut microbiota metabolite IVA inhibits the enrichment of the NuRD complex on the Tph2 promoter to initiate Tph2 transcription, leading to the synthesis of 5-HT in the gut, which subsequently promotes the interaction of HTR1B/1D/1F with Axin1 to activate Wnt/β-catenin signaling. (3) a few CTCs that undergo EMT can survive the blood circulation, and these CTC subpopulations have the CSCs phenotype. Of note, treatment with metronidazole significantly reduced *F. nucleatum* load and inhibiting mCRC, while vancomycin is ineffective against *F. nucleatum* and can even increase its proportion in the intestines of healthy people, destroying the original intestinal environment. The Figure was created with BioRender.com. Abbreviations: 5-HT, 5-hydroxytryptamine; AKT, protein kinase B; CCL, C-C motif chemokine ligand; CEACAM-1; carcinoembryonic antigen-related cell adhesion molecule 1; CRC, colorectal cancer; EGFR, epidermal growth factor receptor; CSCs, cancer stem cells; CTCs, circulating tumour cells; EMT, epithelial-mesenchymal transition; ERK, extracellular signal-regulated kinase; *EVADR*, endogenous retroviral-associated adenocarcinoma long non-coding RNA; FUT8, α1,6-Fucosyltransferase; *F. nucleatum*, *Fusobacterium nucleatum*; HTR, 5-HT receptor; IVA, isovalerate; NETs, neutrophil extracellular traps; NLR, NOD-like receptor; NuRD, nucleosome-remodeling and deacetylase; ROS, oxygen species; TGF-β1, transforming growth factor-β1; TLR4, Toll-like receptor 4; Tph2, tryptophan hydroxylase 2; YBX1, Y-box binding protein 1

In summary, the gut microbiota can promote CRC metastasis by targeting seeds through a number of different mechanisms (Fig. 2). However, given the diversity of the gut microbiota, there are still many unrevealed mechanisms. For example, we do not yet know what virulence factors in different pathogenic bacteria are critical for CRC metastasis, and whether there are other pathogenic bacteria in the gut that collaborate with the main pathogenic bacteria to modify seeds with metastatic potential of CRC. Addressing these questions in future studies will be crucial for a more comprehensive understanding of CRC metastasis mechanisms.

### Promoting changes in the primary TME: targeting the primary soil

The primary TME consists of tumour cells, non-tumour cells, and the surrounding stroma, playing a crucial role in the metastatic cascade response [29]. These cellular and non-cellular factors directly or indirectly stimulate tumour invasion and migration to the vasculature through a variety of mechanisms regulated by chemokines, matrix metalloproteinases (MMPs) and growth factors [29]. The gut microbiota can start the initial stages of CRC metastasis by remodeling the TME [60]. Both myeloid-derived suppressor cells (MDSCs) and tumour-associated macrophages (TAMs) can be recruited by *F. nucleatum* to suppress immunity in CRC patients [61]. Among them, MDSCs have become an important component of TME, which can significantly inhibit T cell activity, with potent immunosuppressive effects and the potential to promote CRC metastasis [62, 63]. Additionally, macrophages have the ability to phagocytose tumour cells alive, leading to the death of tumour cells, which seems to be an important mechanism of tumour immunity [64]. Macrophages can be polarized into M1 or M2 phenotypes due to their high plasticity [65]. Under the influence of specific environmental factors, macrophages may convert to the M2 phenotype during TME reprogramming, contributing to tumour progression through various pathways [60]. Evidence suggests that *F. nucleatum* promotes M2 macrophage polarization through activation of the NF-κB pathway, enabling them to participate in TME reprogramming and stimulate CRC metastasis [66]. Meanwhile, *F. nucleatum* infection significantly increased the proportion of M2 phenotypic macrophages and decreased the proportion of M1 phenotypic macrophages [67]. This leads to impaired phagocytosis of macrophages, promoting CRC progression and metastasis. It is widely recognized that endothelial cells, as a component in the TME, play an important role in CRC metastasis, as the adhesion of spreading CRC cells to endothelial cells is a critical step for extravasation and further distant metastasis, and the adhesion of CRC cells to endothelial cells is also influenced by the gut microbiota [68, 69]. Thus, it is evident that the gut microbiota can promote CRC metastasis by regulating different cells in the TME. Notably, other important cellular components in the TME, such as cancer-associated fibroblasts (CAFs), have also been shown to promote CRC metastasis [70]. However, whether they are regulated by the gut microbiota remains unknown. The relationship between CAFs and the gut microbiota does not seem to be much discussed in the current study.

In addition to the cellular components discussed above, the extracellular matrix (ECM), which is constructed from extracellular macromolecules, serves as a cytoarchitectural scaffold and is a crucial element of the TME [71]. Pro-inflammatory cytokines, chemokines, and adhesion molecules are important components of the ECM, and these metastatic cytokines contribute to the reprogramming of the TME and promote CRC metastasis [72]. It has been demonstrated that gut pathogenic bacteria can lead to a significant increase in the levels of several pro-inflammatory cytokines, such as interleukin (IL)-6, IL-12, IL-9, C-X-C motif chemokine ligand 1 (CXCL1), tumour necrosis factor (TNF)-α, and interferon (IFN)-γ, in the body thereby inducing CRC metastasis [33]. Moreover, the gut microbiota can regulate tumour metabolism and adapt CRC cells to changing environmental conditions, thereby providing environmental support for CRC metastasis [73]. Table 1 lists the specific mechanisms by which the major gut pathogenic bacteria contribute to CRC metastasis by remodelling the TME in multiple ways. Overall, these findings support the notion that the imbalanced gut microbiota can reprogram the TME of CRC by recruiting tumour-infiltrating immune cells, promoting cell adhesion, inducing the secretion of pre-metastatic cytokines, and regulating tumour metabolism, leading to CRC cell migration.

**Table 1:** Mechanisms by which the gut microbiota remolds TME to promote CRC metastasis in different ways

Microbiota	Result	Mechanism	Experimental method	References
*F. nucleatum*	recruit tumour-infiltrating immune cells	*F. nucleatum* can recruit MDSCs and TAMs in TME, causing a decrease in CD8^+^ T cell density to suppress immunity in CRC patients, thereby promoting CRC metastasis.	in vitro	[61]
		*F. nucleatum* induces the expression of the DAMP molecule S100A9 in CRC cells and subsequent M2 macrophage polarization via activating the TLR4/NF-κB signaling pathway.	in vitro	[66]
		*F. nucleatum* infection activates the IL-6/p-STAT3/c-MYC signaling pathway in a TLR4-dependent way to increase M2 macrophage polarization and promote CRC growth and metastasis.	in vitro and in vivo	[67]
		*F. nucleatum* promotes macrophage infiltration through activation of the chemokine CCL20, and induces M2 macrophage polarization, enhancing CRC metastasis.	in vitro	[74]
	promote cell adhesion	*F. nucleatum* can promote adhesion of CRC cells to endothelial cells by inducing the ALPK1/NF-κB/ICAM1 axis, thus promoting extravasation and metastasis.	in vitro and in vivo	[68]
	induce the secretion of pre-metastatic cytokines	*F. nucleatum* drives metastasis by selectively inducing pro-inflammatory and pro-metastatic cytokines IL-8 and CXCL1 from CRC cells via the bacterial surface adhesin Fap2.	in vitro	[75]
		*F. nucleatum* binds to the E-cadherin receptor to cause the activation of β-catenin and stimulates the expression of pro-inflammatory cytokines.	in vivo	[76]
	regulate tumour metabolism	*F. nucleatum* nucleatum increases CRC glycolysis by targeting the lncRNA ENO1-IT1 and the KAT7 histone modification axis, which is an important promoter of CRC metastasis.	in vitro and in vivo	[77, 78]
ETBF	recruit tumour-infiltrating immune cells	ETBF promotes M2 polarization of macrophages, and M2 macrophages can promote CRC metastasis via their secreted proteins and/or regulatory factors.	in vitro	[79]
		The combined effect of BFT and IL-17 on colonic epithelial cells promotes the differentiation of MO-MDSCs that selectively upregulate *Arg1* and *Nos2* to producing NO, inhibiting T cell proliferation.	in vivo	[80]
*E. coli*	recruit tumour-infiltrating immune cells	*E. coli* stimulates the secretion of CTSK, which can bind to TLR4, stimulating M2 polarization of TAMs through an mTOR-dependent pathway and promoting CRC metastasis through the NF-κB pathway.	in vitro and in vivo	[81]

Abbreviations: ALPK1, alpha-kinase 1; *Arg 1*, arginase 1; BFT, *Bacteroides fragilis* toxin; CCL20, C-C motif chemokine ligand 20; CRC, colorectal cancer; CTSK, cathepsin K; CXCL1, C-X-C motif chemokine ligand 1; DAMP, damage-associated molecular pattern; *E. coli*, *Escherichia coli*; ENO1-IT1, enolase1-intronic transcript 1; ETBF, Enterotoxigenic *Bacteroides fragilis*; *F. nucleatum*, *Fusobacterium nucleatum*; ICAM1, intercellular adhesion molecule 1; IL, interleukin; KAT7, lysine acetyltransferase 7; lncRNA, long non-coding RNA; MDSCs, myeloid-derived suppressor cells; MO-MDSCs, monocytic-MDSCs; mTOR, mammalian target of rapamycin; NF-κB, nuclear factor kappa B; NO, nitric oxide; *Nos*2, inducible nitric oxide synthase 2; p-STAT3, phosphorylated signal transducer and activator of transcription 3; TAMs, tumour-associated macrophages; TLR4, Toll-like receptor 4; TME, tumour microenvironment

### Promoting PMN formation: targeting the secondary soil

**Fig. 3:**
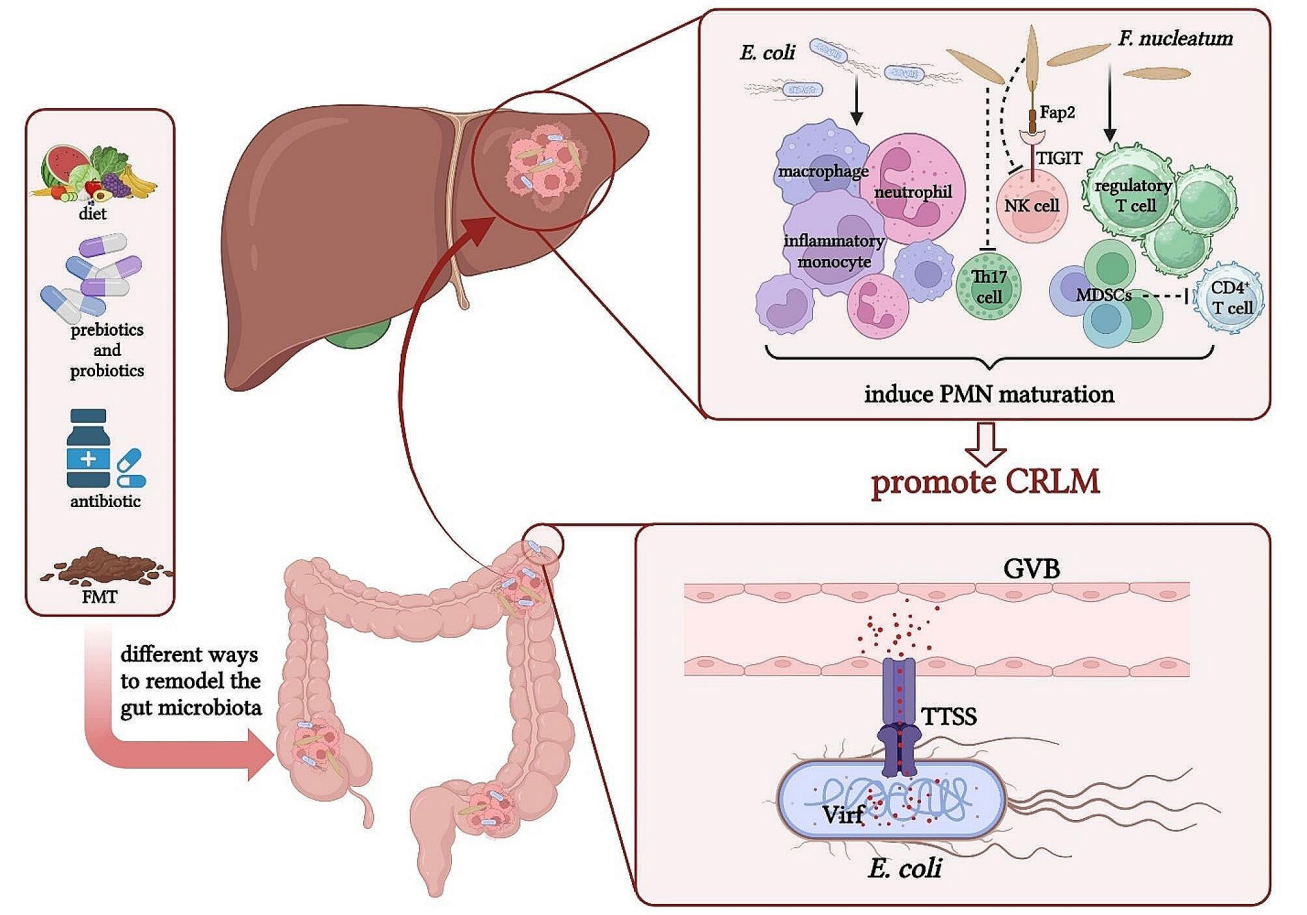
The mechanisms by which the gut microbiota promotes CRC metastasis by targeting the secondary soil. *E. coli* can directly open the GVB through a TTSS Virf-dependent mechanism and translocate into the liver, where it initiates the recruitment of macrophages, neutrophils, and inflammatory monocytes, which promotes PMN maturation and favors mCRC formation. *F. nucleatum* significantly increases plasma levels of pro-inflammatory cytokines as well as modulates liver immune responses, including the recruitment of MDSCs, decreased infiltration of NK cells and Th17 cells, and increased accumulation of regulatory T cells. The combination of Fap2 protein of *F. nucleatum* and NK Cell receptor, T cell immunoreceptor with immunoglobulin and ITIM domain (TIGIT), can protect tumours from NK cells attack, thus benefiting the survival of CRC cells. *F. nucleatum* increases the expression of MDSCs to inhibit CD4^+^ T cells. All these mechanisms result in CRLM. The Figure was created with BioRender.com. Abbreviations: CRLM, colorectal cancer liver metastasis; *E. coli*, *Escherichia coli*; *F. nucleatum*, *Fusobacterium nucleatum*; GVB, gut vascular barrier; mCRC, metastatic colorectal cancer; MDSCs, myeloid-derived suppressor cells; NK, natural killer; PMN, pre-metastatic niche; Th17, T helper-17; TTSS, type III secretion system; Virf, virulence factor

In order to successfully sow the surviving seeds into the secondary soil, the primary soil will secrete tumour-derived factors and extracellular vesicles (EVs) prior to the spread of tumour cells to remodel the potential site into a metastasis-friendly environment, which is referred to as the PMN, i.e., the secondary soil, which consists of many elements that influence tumour metastasis and plays a key role in promoting tumour cells colonization and metastasis [29]. There is evidence to suggest that an important reason why CRC metastasis preferentially grows in the liver is that the liver contains specific cellular and molecular components that stimulate PMN formation, which synergize to build an immunosuppressive and inflammatory microenvironment, facilitating the extravasation, invasion, and colonization of CRC cells [20]. Specifically, PMN formation in the liver will undergo a series of changes, including activation of hepatic stellate cells (HSCs), overexpression of pro-inflammatory cytokines, hypoxia, regulation of metabolism, recruitment of MDSCs, enhancement of vascular permeability, and angiogenesis [82–87]. All of these alterations favor colonization of CRC cells, and it has been found that the gut microbiota can migrate to the liver through certain pathways to induce some of the above factors to promote liver PMN formation [33, 34] (Fig. 3). *E. coli* can directly open the gut vascular barrier (GVB) through a type III secretion system (TTSS) virulence factor (Virf)-dependent mechanism and then translocate into the liver, where it can initiate the recruitment of innate immune cells, thereby triggering PMN maturation and facilitating mCRC formation [34]. The innate immune cells that are recruited mainly include macrophages, neutrophils and inflammatory monocytes [34]. However, the reasons why these innate immune cells become accomplices in tumour growth and metastasis as well as the underlying mechanisms still need to be further explored. Notably, the GVB is an anatomical structure that controls bacterial dissemination along the gut-liver axis, which refers to the bi-directional relationship between the gut along with its microbiota and the liver [34, 88]. *F. nucleatum* has also been found to affect liver immunity via the gut-liver axis: when mice were orally administered *F. nucleatum*, plasma levels of pro-inflammatory cytokines were significantly increased and the liver immune response was modulated, such as the recruitment of MDSCs, reduced infiltration of natural killer (NK) cells and T helper-17 (Th17) cells, and increased accumulation of regulatory T cells, resulting in significantly enhanced CRLM [33]. It is worth noting that the regulatory mechanism of *F. nucleatum* on NK cells is that the combination of Fap2 protein of *F. nucleatum* and NK Cell receptor, T cell immunoreceptor with immunoglobulin and ITIM domain (TIGIT), can protect tumours from NK cells attack, thereby benefiting the survival of CRC cells [51]. Although the gut microbiota has been shown to activate HSCs to cause liver diseases, it has not been directly demonstrated that the imbalanced gut microbiota promotes CRLM by inducing the activation of HSCs [89]. Therefore, the relationship between the gut microbiota and liver PMN maturation deserves further confirmation and exploration. Furthermore, in addition to liver PMN, there is a lack of research on whether and how the imbalanced gut microbiota can regulate PMN at other CRC metastatic sites, and the mechanisms by which the gut microbiota reaches these sites remain unclear. These may be hot topics for future research.

## Targeting the gut microbiota for inhibiting CRC metastasis: clinical approaches for prevention, treatment, and prognostic evaluation

From the preceding discussion, it is clear that the imbalanced gut microbiota can promote CRC metastasis through various mechanisms outlined in the “seed and soil” hypothesis. Therefore, targeting the gut microbiota emerges as a promising clinical strategy for preventing and supplementing the treatment of mCRC, as well as assessing patient prognosis.

As previously noted, mCRC patients have a severely disordered gut microbiota, and therefore, restoring the gut homeostasis is beneficial in impeding the process of metastasis, which may be achieved by using probiotic supplements [90, 91]. However, it should be noted that it is hard to judge the efficiency of oral probiotics in colonizing the gut, and it is unknown whether excessive probiotic colonization will also cause imbalance in the gut microbiota, so the optimal dosage of oral probiotics needs to be further discussed. Furthermore, prebiotics are nondigestible and selectively fermented dietary fibers that can serve as a substrate for the gut microbiota to promote the metabolism of lipids, proteins, and minerals and to produce metabolites that may protect against the intestinal function [92]. Frequently reported prebiotics include fructooligosaccharides, galactooligosaccharides and inulin [92]. Studies demonstrate that synbiotics (combinations of prebiotics and probiotics) can lead to the downregulation of genes involved in pro-cancer pathways [93]. These findings underscore the substantial potential of probiotics and prebiotics in mCRC prevention.

In addition, the gut microbiota can influence the clinical outcome of systemic therapies. It was found that mCRC patients with higher diversity of the gut microbiota responded well to the combination of chemotherapy and targeted therapy [13]. Table 2 summarizes clinical and preclinical studies targeting the gut microbiota in the adjuvant treatment of mCRC. However, these treatments still have some drawbacks. Among the challenges associated with these interventions, oral probiotics present limitations, as previously mentioned. Furthermore, the use of antibiotics poses its own set of issues. Although antibiotics can efficiently eliminate harmful bacteria, their indiscriminate action may disrupt the delicate balance of the homeostatic environment of the gut microbiota [94]. Hence, the judicious use of targeted antibiotics aimed at specific pathogenic bacteria, whose levels are abnormally elevated, is imperative for successful treatment. Besides, patients receiving fecal microbiota transplantation (FMT) are at risk of life-threatening infections due to the presence of potential pathogenic bacteria in the donor’s stool, so the safety of FMT needs to be further validated [95]. The findings from these studies underscore the potential of integrating gut microbiota with conventional clinical approaches for managing mCRC. Nevertheless, expediting the translation of basic scientific research into clinical application constitutes the primary objective of our work. Therefore, urgent initiation of additional clinical trials is imperative to substantiate the efficacy of these foundational experiment. Table 3 lists the current ongoing clinical trials of microbiota-associated mCRC.

The gut microbiota can also serve as a prognostic tool for mCRC patients. Irinotecan, a broad-spectrum anti-tumour drug commonly used in mCRC treatment, frequently induces delayed-onset diarrhoea. This adverse effect is attributed to the activity of the gut bacterium β-glucuronidase (BGUS), and administration of a selective BGUS inhibitor mitigates irinotecan-induced diarrhoea [96]. Consequently, the expression of BGUS and its enzymatic activity in gut microbiota can potentially predict the severity of chemotherapy-related side effects [96]. Additionally, a decline in beneficial bacteria, an upsurge in pathogenic bacteria, and perturbations in gut microbiota homeostasis observed during treatment signify, to a certain extent, an unfavourable therapeutic outcome. Notably, *F. nucleatum* is identified as an independent adverse prognostic indicator for mCRC [38]. These findings demonstrate an important role of the gut microbiota in assessing therapeutic response and prognosis in patients with mCRC.

Taken together, the gut microbiota has great potential for clinical application, but given the aforementioned dilemmas of targeting the gut microbiota in the prevention and adjuvant treatment of mCRC, other more effective precision therapies manipulating the gut microbiota need to be developed as soon as possible with the aim of clinically mitigating mCRC and improving the quality of life of mCRC patients.

**Table 2:** Applications of targeting the gut microbiota in the adjuvant therapy of mCRC

Microbiota intervention	Cancer Therapy	Outcome	Research type	References
probiotics (oral C.B)	5-FU and anti-PD-1	attenuating 5-FU resistance and enhancing anti-PD-1 immunotherapy to inhibit CRC cells proliferation and metastasis	zoopery	[97]
probiotic mixture	not applicable	reducing angiogenesis and inhibiting CRLM	zoopery	[91]
probiotic mixture	surgery	changing the gut microenvironment to cause to a decrease in pro-inflammatory cytokines, improving postoperative survival	clinical trail	[98]
antibiotics (gentamicin and amikacin)	not applicable	inhibiting CRLM in a mouse model	zoopery	[99]
antibiotics (minocycline)	not applicable	impeding the EMT process in CRC cells to inhibit metastasis	zoopery	[100]
FMT	anti-PD-1 and anti-VEGF	reducing the size of the tumour to a level suitable for surgical resection and achieving complete pathological remission after surgery	case report	[101]
HFD	OXP and 5-FU	synergistically enhancing the treatment effect of chemotherapeutics for CRC peritoneal metastasis	zoopery	[102]

Abbreviations: C.B, *Clostridium butyricum*; CRLM, colorectal cancer liver metastasis; EMT, epithelial-mesenchymal transition; FMT, fecal microbiota transplantation; HFD, high-fat diet; OXP, oxaliplatin; PD-1, programmed cell death protein-1; VEGF, vascular endothelial growth factor; 5-FU, 5-fluorouracil

**Table 3:** The ongoing clinical trials of microbiota-associated metastatic colorectal cancer

NCT number	Status	Interventions	Phases	Purposes
NCT04729322	not recruiting	procedure: biopsy, FMT; drugs: FMT capsule, metronidazole, neomycin, vancomycin; biological: nivolumab, pembrolizumab; Other: questionnaire	Phase 2	adjuvant treatment: evaluate the efficacy of pembrolizumab or nivolumab in conjunction with FMT
NCT03941080	recruiting	diagnostic test: fecal sample, blood sample; behavioral: questionnaire	not applicable	adjuvant treatment: study the relation between the gut microbiome and the effects of chemotherapy
NCT04131803	not yet recruiting	drugs: Bifico (also known as “bifidobacterium trifidum live powder”, a probiotic preparation) combined with chemotherapy plus targeted therapy, chemotherapy plus targeted therapy	not applicable	adjuvant treatment: assess Bifico combined with standard chemotherapy plus targeted therapy compared to standard chemotherapy plus targeted therapy for efficacy and safety of metastatic colorectal cancer
NCT06049901	recruiting	drug: nitazoxanide	Phase 3	adjuvant treatment: evaluate the efficacy and safety of nitazoxanide
NCT05655780	recruiting	not applicable	not applicable	judge prognosis: find out biomarkers to predict response and side effects during irinotecan treatment

Abbreviations: FMT, fecal microbiota transplant

## Conclusion

Tumour metastasis presents a significant challenge in treating CRC and remains a primary factor in reducing patient survival rates. Recent studies have increasingly indicated the involvement of the gut microbiota in various stages of CRC metastasis. To deepen our understanding of CRC metastasis and facilitate the development of targeted therapeutic strategies against specific microbial species, a thorough investigation of the mechanisms through which an imbalanced gut microbiota promotes CRC metastasis is essential. The vast diversity of the gut microbiota complicates the identification of individual microorganisms’ mechanisms; however, considering the gut microbiota as a whole, exploring potential interactions among its components and the convergence of various mechanisms influencing CRC metastasis is crucial. Moreover, investigating the impact of gut microbiota metabolites on mCRC is warranted.

Given the manipulability of the gut microbiota, it represents an attractive therapeutic target for mCRC patients. Several current approaches for modulating the gut microbiota have demonstrated adjuvant therapeutic potential for mCRC. Nonetheless, these techniques are constrained by their limited clinical effectiveness. Further research is essential to expedite their translation into clinical practice. Future strategies for gut microbiota-targeted therapy may focus on the following key areas: (1) Designing rational drug delivery pathways with the goal of releasing various microbiota-associated therapeutic agents precisely and under control, thereby minimizing microbiota exposure to non-target tissues or organs to prevent the imbalance of microbiota at these sites. (2) Combining gut microbiota modulation with conventional chemotherapy, radiotherapy, or immunotherapy in order to concurrently target multiple metastasis-related pathways, improving the efficacy of conventional treatment. (3) Developing technologies and models for dynamically monitoring changes in the gut microbiota of mCRC patients to enable precise and personalized treatment strategies, considering the significant inter-individual variation in gut microbiota composition.

## Data Availability

No datasets were generated or analysed during the current study.

## Abbreviations

5-HT: 5-hydroxytryptamine
AKT: Protein kinase B
BGUS: β-glucuronidase
*B. fragilis*: *Bacteroides fragilis*
CAFs: Cancer-associated fibroblasts
CCL: C-C motif chemokine ligand
CEACAM-1: Carcinoembryonic antigen-related cell adhesion molecule 1
CRC: Colorectal cancer
CRLM: CRC liver metastasis
CRS: Cytokine release syndrome
CSCs: Cancer stem cells
CTCs: Circulating tumour cells
CXCL1: C-X-C motif chemokine ligand 1
Dps: DNA hunger/stationary phase protective proteins
ECM: Extracellular matrix
EGF: Epidermal growth factor
EGFR: Epidermal growth factor receptor
EMT: Epithelial-mesenchymal transition
ERK: Extracellular signal-regulated kinase
ETBF: Enterotoxigenic *B. fragilis*
*EVADR*: Endogenous retroviral-associated adenocarcinoma lncRNA
EVs: Extracellular vesicles
*E. coli*: *Escherichia coli*
FMT: Fecal microbiota transplantation
FUT8: α1,6-Fucosyltransferase
*F. nucleatum*: *Fusobacterium nucleatum*
*F. prausnitzii*: *Faecalibacterium prausnitzii*
GVB: Gut vascular barrier
HSCs: Hepatic stellate cells
HTR: 5-HT receptor
IFN: Interferon
IL: Interleukin
IVA: Isovalerate
lncRNAs: Long non-coding RNAs
LSECs: Liver sinusoidal endothelial cells
mCRC: Metastatic CRC
MDSCs: Myeloid-derived suppressor cells
miRNAs: MicroRNAs
MMPs: Matrix metalloproteinases
NETs: Neutrophil extracellular traps
NK: Natural killer
NLR: NOD-like receptor
PMN: Pre-metastatic niche
ROS: Reactive oxygen species
*S. enterica*: *Salmonella enteric*
TAMs: Tumour-associated macrophages
TGF-β1: Transforming growth factor-β1
Th17: T helper-17
TIGIT: T cell immunoreceptor with immunoglobulin and ITIM domain
TLR: Toll-like receptor
TME: Tumour microenvironment
Tph2: Tryptophan hydroxylase 2
TTSS: Type III secretion system
VEGF: Vascular endothelial growth factor
Virf: Virulence factor
YBX1: Y-box binding protein 1
